# High Prevalence of Methicillin-Resistant *Staphylococcus aureus* among Healthcare Facilities and Its Related Factors in Myanmar (2018–2019)

**DOI:** 10.3390/tropicalmed6020070

**Published:** 2021-05-06

**Authors:** Pan Ei Soe, Wai Wai Han, Karuna D. Sagili, Srinath Satyanarayana, Priyanka Shrestha, Thi Thi Htoon, Htay Htay Tin

**Affiliations:** 1 National Health Laboratory, Ministry of Health and Sports, Yangon 11191, Myanmar; thithitun@mohs.gov.mm (T.T.H.); htayhtaytin@mohs.gov.mm (H.H.T.); 2 Medical Statistics Division, Department of Medical Research, Ministry of Health and Sports, Yangon 11191, Myanmar; waiwaihan@mohs.gov.mm; 3 International Union Against Tuberculosis and Lung Disease, South East Asia Office, New Delhi 110016, India; ksagili@theunion.org (K.D.S.); ssrinath@theunion.org (S.S.); 4 WHO Health Emergencies Programme, Kathmandu 44600, Nepal; priyankasth23@gmail.com

**Keywords:** antimicrobial resistance, hospital infections, MRSA, Gram-positive bacteria, SORT IT, operations research

## Abstract

Background: Antimicrobial resistance (AMR) is a growing global health problem. *Staphylococcus aureus* (*SA*) is a common bacterium associated with a variety of community and hospital infections. Methicillin-resistant *Staphylococcus aureus* (MRSA) accounts for most *SA* related morbidity and mortality. In this study, we determined the prevalence and factors associated with *SA* and MRSA in Myanmar. Methods: We collected the data retrospectively by reviewing an electronic register containing the results of bacterial culture and antibiotic susceptibility testing of biological specimens received from healthcare facilities during 2018–2019. Results: Of the 37,798 biological specimens with bacterial culture growth, 22% (8244) were Gram-positive. Among the Gram-positive bacteria, 42% (2801) were *SA*, of which 48% (1331) were judged as MRSA by phenotypic methods. The prevalence of MRSA was higher in the older age groups, in female patients, in urine specimens and specimens received from the intensive care unit and dermatology departments. One site (Site F) had the highest MRSA prevalence of the seven AMR sentinel sites. Most *SA* isolates were sensitive to vancomycin (90%) by phenotypic methods. Conclusions: The high prevalence of MRSA indicates a major public health threat. There is an urgent need to strengthen the AMR surveillance and hospital infection control program in Myanmar.

## 1. Introduction

Antimicrobial resistance (AMR) is a serious emerging global health problem in this century. *Staphylococcus aureus* (*SA*) is an antibiotic-resistant pathogen of significant public health concern [1]. Humans can become infected with *SA* both in the community and in healthcare settings. *SA* causes a wide range of human infections like bacteremia, endocarditis, skin and soft tissue infections, bone and joint infections and hospital-acquired infections [2].

The inappropriate use of antibiotics contributes to antibiotic resistance in *SA*. MRSA (methicillin-resistant *Staphylococcus aureus*) is a subgroup of *SA*. As the name suggests, MRSA does not respond to common antibiotics, such as methicillin, amoxicillin, and penicillin. In the United States, the incidence of MRSA bloodstream infections declined from 74% to 40% during 2005–2016. Despite this decline, it is estimated that nearly 120,000 *SA* bloodstream infections and 20,000 *SA*-associated deaths occurred in 2017 [3]. The Asia Pacific Regional Resistance Surveillance program reported that 26% to 73% of *SA* isolates from healthcare settings in the region were resistant to methicillin [4].

Infection with MRSA remains associated with poorer clinical outcomes and increased healthcare costs. A multicenter study conducted in China between 2013 and 2015 showed that the MRSA infection was significantly associated with higher total hospital cost, longer length of hospital stay, and increased mortality rate as compared to Methicillin-sensitive *Staphylococcus aureus* (MSSA) infection, especially in patients with underlying diseases such as malignancy or chronic pulmonary diseases [5]. However, evidence from high-income countries proved that implementing an effective hospital infection control program significantly reduces the morbidity and mortality of MRSA-associated infections [6,7,8]. The improvement in hand hygiene compliance can significantly decrease MRSA rates in hospitals [9].

According to the World Health Organization (WHO) estimate, Myanmar has the highest MRSA proportion (26%) among the South East Asian countries that reported national data relating to antibiotic resistance. However, this estimate was based on approximately 30 isolates only [1]. To date, there is minimal information on the prevalence of MRSA, morbidity, and mortality in Myanmar from a few publications on microbiological and animal studies [10,11,12]. A recently published study conducted in a tertiary care hospital in Myanmar revealed that the molecular detection of MRSA accounted for 13.8% [13]. A retrospective study from one hospital examining blood culture results showed a decline in MRSA among *SA* isolates (38.7% to 18.8%) over eight years [14]. Establishing a proper surveillance system and an effective hospital infection control system is mandatory to minimize the emergence of MRSA and to reduce its spread. Although national guidelines on hospital infection control were developed in Myanmar in 2016, clinicians’ adherence to the guidelines is still low [15]. Besides, guidelines related to antibiotic prescription do not exist at the national level, although some tertiary hospitals have developed their own antibiotic guidelines. This study aimed to determine the prevalence of *SA* and the factors associated with MRSA in healthcare settings in Myanmar during 2018–2019. The objectives of the study were (1) to assess the number (and proportion) of samples with *SA* infection among the total biological samples received for bacterial culture and drug susceptibility testing at seven AMR sentinel sites between 2018 and 2019; (2) to describe the antibiotic susceptibility pattern of *SA* infection and assess the number and proportion with MRSA infection; (3) to describe the demographic and clinical profile of patients and determine their association with MRSA.

## 2. Material and Methods

### 2.1. Study Design

This was a retrospective descriptive study based on the electronic register record of seven AMR sentinel laboratories in Myanmar.

### 2.2. Setting

#### 2.2.1. General Setting

The Union of the Republic of Myanmar is located in the South East Asian region and bordered by the Bay of Bengal, Andaman Sea, Gulf of Thailand, and the countries of Bangladesh, India, China, Laos, and Thailand. The country is administratively divided into 14 States/Regions and Nay Pyi Taw Union Territory. It has a population of 51.48 million. Healthcare is provided by both the public and private sectors. General practitioner clinics and drug shops are the initial points of healthcare seeking for most populations. Antibiotics are readily available over the counter.

#### 2.2.2. Specific Setting

**Figure 1:**
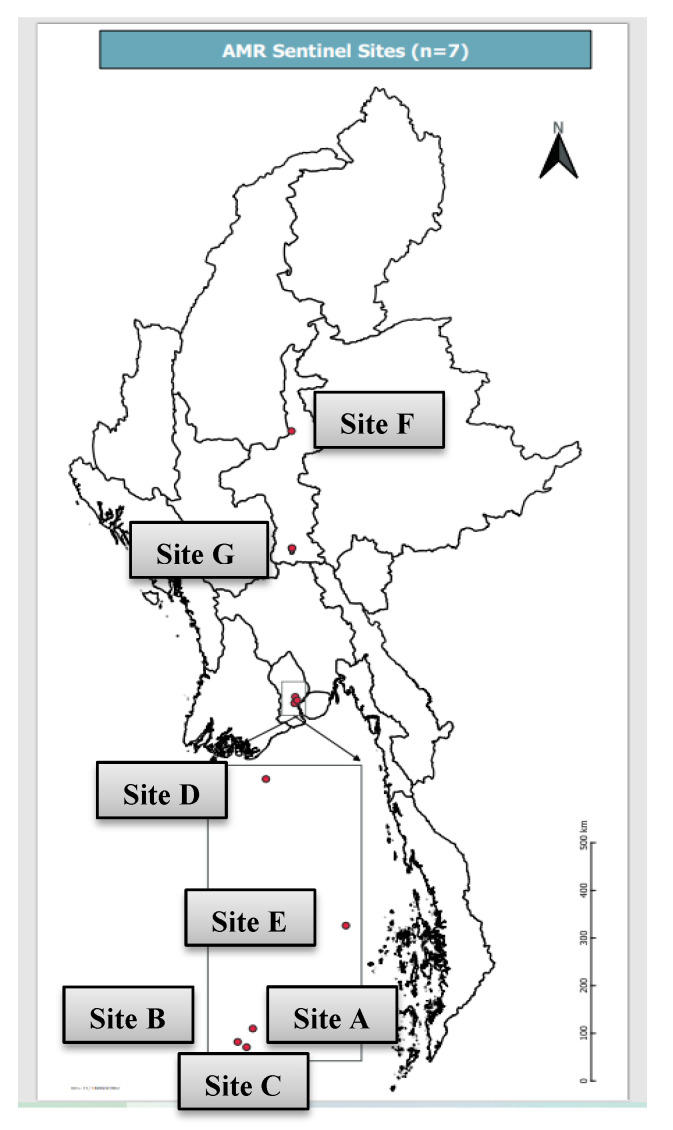
Distribution of seven AMR sentinel sites in Myanmar.

In Myanmar, the AMR surveillance system at the national level is being carried out through seven public hospitals and laboratories, which can cover AMR’s overall situation in Myanmar. Five of them (Site A, B, C, D and E) are located in Yangon Region, covering the Yangon Region population and some population from the lower part of Myanmar. Site F is located in the Mandalay Region, and it covers the population from upper Myanmar. Site G is located in Nay Pyi Taw Union Territory in the central part of Myanmar, and it covers the population in Nay Pyi Taw Union Territory and surrounding townships. The populations of Yangon Region and Mandalay Region are 7.3 million and 6.1 million, respectively. The distribution of the seven sentinel sites is shown in Figure 1.

#### 2.2.3. AMR Surveillance in Myanmar

The National Action Plan (NAP) to combat AMR has been developed in line with the Global Action Plan of AMR since 2017, with five strategic objectives (awareness, surveillance, infection prevention and control, antimicrobial usage, and research and innovation). National Multi-sectoral Steering Committee (NMSC) was organized to provide the necessary political commitments to fight against AMR. Five technical working groups (TWGs) were constituted under NMSC to implement the five strategic objectives of NAP AMR. The National Coordinating Centre (NCC) for AMR is responsible for coordinating between the NMSC and the five TWGs for combating AMR. Myanmar developed National AMR Surveillance Guidelines (draft) in 2020 for standardization of the AMR surveillance system.

#### 2.2.4. Laboratory Procedure

Patients’ clinical specimens were collected and processed by using standard microbiological procedures. The first- and second-line drugs and antibiotic susceptibility testing of these drugs were conducted according to the Clinical and Laboratory Standards Institute (CLSI) guidelines [16]. MRSA was screened for phenotypically by using oxacillin MIC (≥4 µg/mL) in an automated system and cefoxitin disc (30 µg) for manual method. MIC determinations by broth or agar dilution or by E test using a 0.5 McFarland standard to prepare inoculum are the gold standard for determining vancomycin susceptibility. In this study, there was no standard method used for VRSA detection since the sentinel sites did not have this capacity. Vancomycin sensitivity testing was done by only automated Vitek 2 system for clinical purposes and vancomycin MIC ≥ 16 is interpreted as resistant. Antibiotic susceptibility pattern was detected by using the modified Kirby–Bauer method or Vitek 2 AST GP 67 according to the capacity of each sentinel sites. Due to the resource constraint, some sentinel sites (especially Site F) used manual method and some used automated Vitek 2 system. In this study 70% of samples from all sentinel sites were tested by Vitek 2 automated system and the rest were tested by manual method. The reference ranges of drug susceptibility pattern were set as shown in Table 1. The testing validity of all sentinel sites were evaluated by regular internal quality control and national external quality assessment scheme (EQAS) of national reference laboratory. The routine culture and sensitivity data were recorded both in the register book and electronic database WHONET software which is a free desktop Windows application for the management and analysis of microbiology laboratory data with a particular focus on antimicrobial resistance surveillance developed and supported by the WHO Collaborating Centre for Surveillance of Antimicrobial Resistance The AMR data from all sentinel sites were sent to the National Coordinating Centre (NCC) bi-annually. NCC combines all data and validates and then annually uploads it to the Global Antimicrobial Surveillance System (GLASS) IT platform. National Health Laboratory is responsible for quality control of laboratories throughout the country as an organizer and provider of national external quality assessment scheme (NEQAS) of culture and sensitivity testing bi-annually.

### 2.3. Study Population and Period

The study populations were all patients’ specimens sent for routine culture and antibiotic susceptibility testing in seven AMR sentinel sites during the study period of 1 January 2018 to 31 December 2019.

### 2.4. Data Variables and Sources of Data

Data variables included specimen ID, lab test registration date, sentinel sites, number of the specimen, demographic data, type and source (by ward) of the patient whose specimen was sent for culture and sensitivity, specimen type, culture result and antibiotic susceptibility pattern.

Routine laboratory register data at seven sentinel sites were maintained in an electronic database (WHONET software) starting from 2018. Data on different variables were entered routinely into the WHONET software from paper-based laboratory registers by technicians from each sentinel laboratory. We obtained data from the WHONET database of NCC and laboratory registers of seven sentinel sites.

### 2.5. Data Collection, Analysis and Statistics

Data from WHONET software was extracted into Microsoft Excel and imported into EpiData Analysis and Stata Statistical Software. We described the prevalence of *SA*, the antibiotic susceptibility pattern and MRSA in numbers and proportions.

The patient characteristics of those with *SA* infection are described in numbers and proportions. The associations between patient characteristics and the presence of MRSA among their specimens with *SA* infection are described by prevalence ratios and adjusted prevalence ratios. We used bi-variable and multivariable binomial log models for obtaining the prevalence and adjusted prevalence ratios. A *p*-value < 0.05 has been considered for statistical significance.

## 3. Results

### 3.1. Culture and Sensitivity of SA

**Figure 2:**
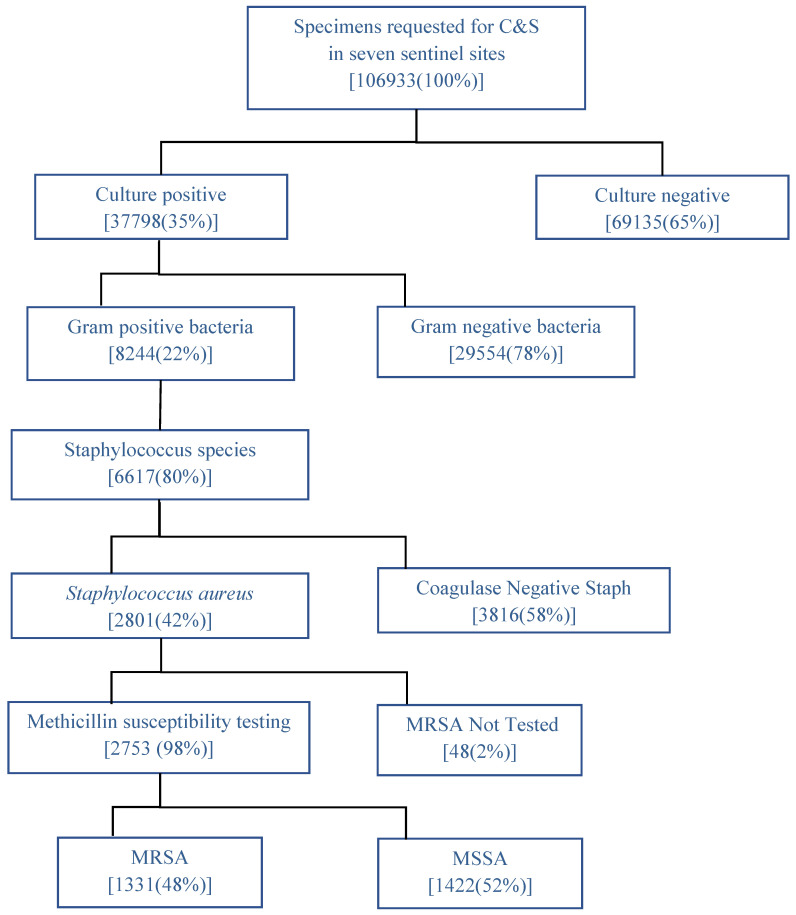
Results of culture and drug susceptibility testing of samples received at seven AMR sentinel sites in Myanmar in 2018–2019 and the prevalence of *Staphylococcus aureus* and methicillin-resistant *Staphylococcus aureus.* C&S = culture and sensitivity. MRSA = Methicillin-resistant *Staphylococcus aureus.* MSSA = Methicillin sensitive *Staphylococcus aureus.* AMR = Antimicrobial Resistance.

Figure 2 shows the results of culture and drug susceptibility testing of samples received at seven AMR sentinel sites in Myanmar in 2018–2019 and the prevalence of *SA* and MRSA. During 2018 and 2019, a total of 106,933 specimens were received for culture and sensitivity in the seven sentinel sites, of which Gram-positive bacterial growth was observed in 8244 samples. Nearly 80% of the Gram-positive bacteria belonged to the Staphylococcus species. Among the Staphylococcus species, 2801 were *SA*, of which 1331 (48%) were MRSA.

### 3.2. Antibiotic Susceptibility Pattern of SA

The antibiotic susceptibility pattern of *SA* isolated in seven AMR Sentinel Sites is described in Table 2. Among 2753 *SA* isolates, 48% were found to be MRSA. The first-line and second-line drugs were grouped according to the Clinical Laboratory Standard Institute guideline. The most sensitive first-line drug was nitrofurantoin (97%), and the least sensitive first-line drug was penicillin (3%). Among the second-line drugs, high sensitivity was observed to Linezolid (91%), and high resistance was seen to tetracycline (58%).

### 3.3. Distribution of SA Infection among the Isolates

The demographic and clinical characteristics of patients whose isolates tested positive for *SA* infection in seven AMR Sentinel Sites are described in Table 3. The total number of isolates with *SA* infection among Gram-positive isolates in 2018 and 2019 were 1324 (35%) and 1477 (34%), respectively. In 2018 and 2019, *SA* infection was mainly found in the 15–44 years age group (about 38%). It was mainly found in the isolates of male patients (54%) and hospitalized patients (79% & 90% in respective years). The most common specimens associated with *SA* infection were wound/pus and blood.

### 3.4. Prevalence of SA and MRSA in Seven AMR Sentinel Sites

The prevalence of *SA* and MRSA in seven sentinel sites during 2018 and 2019 is shown in Table 4. The highest proportion of *SA* and MRSA were found in Site F. The lowest proportion of *SA* was found in Site B, while Site G had the lowest proportion of MRSA in both study years. There was a considerable decrease in the proportions of *SA* and MRSA in Site E in 2019.

### 3.5. Factors Associated with MRSA

Demographic and clinical factors associated with MRSA infection in seven AMR sentinel sites, Myanmar, are described in Table 5. The factors that were associated with a higher prevalence of MRSA were: year of sample collection (higher prevalence in 2018 when compared to 2019), age (higher prevalence in age groups 15 and above), gender (higher prevalence in females when compared to males), type of specimen (higher prevalence in urine when compared to blood), source of the patient (higher prevalence in those from ICU and dermatology when compared to medical wards), and the sentinel laboratory site (higher prevalence in Site F) when compared to all other sites.

## 4. Discussion

This study revealed three important findings on the prevalence of *SA*, MRSA and their associated factors. First, there was no significant difference in *SA* prevalence among isolates between 2018 and 2019; however, there was a decrease in MRSA prevalence in 2019. Second, the antibiotic susceptibility pattern showed *SA* isolates were highly resistant to a variety of first-line antibiotics (penicillin, erythromycin) and second-line antibiotics (tetracycline, gentamicin, ciprofloxacin, levofloxacin). Almost all samples of *SA* were resistant to penicillin but were highly sensitive to nitrofurantoin. Third, MRSA infection was significantly associated with gender, age, specimen types, source of patients and sentinel sites.

The prevalence of *SA* infection among biological specimens sent for culture and drug susceptibility testing in this study were five times higher than the results reported in the previous two studies from Myanmar, both of which were conducted in a single hospital and only focused on blood specimens [17,18]. Our study also showed a higher *SA* prevalence than the studies undertaken in India and Nepal [19,20]. The reasons for the high prevalence need to be addressed. The national guidelines on infection prevention and control were launched in 2016. The Ministry of Health and Sports Myanmar allocated the required budget for hospitals to implement the infection control activities and provided the necessary training. Nevertheless, our study results indicate that infection control efforts should go beyond establishing guidelines, budget allocation, and training. On the other hand, limited human resources and poor hospital infrastructure may have led to the lax implementation of infection prevention and control measures.

Studies across Asia showed a wide range of methicillin resistance among *SA* isolates with higher rates in hospital settings (0.7% to 75%) and lower rates in community settings (0% to 48%) [21,22,23,24]. This is perhaps due to the differences in the study population and geographic locations where the studies were conducted. Previous studies have shown that the prevalence of MRSA among *SA* is highly variable. According to the Annual NHL report, there was a rise in MRSA prevalence in Myanmar from 30% in 2016 to 44% in 2019; however, our study observed a significant decline in 2019 (0.78–0.91; *p* < 0.001) compared to 2018 [25,26]. This reduction may be likely due to raised AMR awareness among healthcare providers, improved testing capacity of laboratories and diagnostic stewardship activities. The lower percentage in MRSA was found in two studies performed in Myanmar in which the MRSA prevalence was 8% and 13.8%, respectively [10,13]. The reason is due to the differences in detection methodology which use mecA gene PCR for MRSA detection and the focus on only one hospital.

Resistance to the penicillin group of antibiotics, erythromycin and gentamycin, among MRSA isolates has been reported in two previous studies in Myanmar, one of which reviewed hospital records since 2005 [10,14]. However, Myanmar-based studies determining drug sensitivity for second-line antibiotics among MRSA are scant, and our study is the first one to report on this. Our study indicates high levels of resistance to second-line antibiotics. Over the counter availability and widespread use of penicillin group of antibiotics among the general practitioners and community may be responsible for high levels of resistance to these drugs. A similar antibiotic susceptibility pattern for second-line antibiotics was found in a study in Pakistan [27] but it was quite different from a study undertaken in India [28]. When reviewing the global prevalence of VRSA in a meta-analysis study, the overall percentage of VRSA increased from 1.2% to 2.4% over a 10 year duration [29]. In this study, the percentage of vancomycin-resistant *SA* (VRSA) is quite high (8%). This is an important indication that vancomycin testing in Myanmar should be standardized and required confirmation to get more reliable and correct data. Further focused research on VRSA prevalence in Myanmar is recommended.

Our study found that *SA* infection was common among male patients, while MRSA infection was significantly associated with the female gender. The effect of gender on *SA* infection is unknown. It is speculated that female sex hormones may modify the immune response and impact contracting infection [30].

Our study observed a positive association between MRSA infection and age [31], and a systematic literature review from India also showed similar evidence [32]. Older age groups are more prone to get infected due to decreased host resistance and increased exposure to healthcare settings. In contrast to other studies, we found the highest proportion of MRSA in urine specimens [23,33,34]. This may be due to urinary catheterization practice and the colonization by MRSA of indwelling urinary catheters.

We identified that certain units of the hospital (ICU and dermatological wards) and in particular the laboratory site (Site F) were significantly associated with a higher rate of MRSA infection. The reason for the significantly higher rate of MRSA needs to be addressed. As a first opinion, there may really be a higher MRSA prevalence in the population, especially at site F. The second probable reason is the methods used for MRSA detection that are the phenotypic method (automated Vitek 2 system or manual method by modified Kirby–Bauer method) by cefoxitin disc (30 µg); site F used only manual methods. Detailed evaluation of the situation related to site F will be considered for future research. The phenotypic method could be influenced by various factors, e.g., temperature and period of incubation, salt concentration in the media and the potency of the antibiotic disc. In such a case, the results of susceptibility testing by phenotypic methods may show generally higher resistance rate. Therefore, the quality of the antibiotic disc and methodology need to be validated. Moreover, the infrastructure of wards and hospitals, the effective implementation of infection control activities, and having patients with a high proportion of community-acquired MRSA are the possible factors influencing such an association [31]. Additionally, the lack of screening among healthcare providers for MRSA infection could also lead to higher MRSA prevalence in hospitals [35].

The strengths of this study are: (a) the use of nationwide data from all sentinel sites has increased the representativeness of the study results; (b) this is the first study assessing antibiotic susceptibility pattern of *SA* and MRSA in both first- and second-line antibiotics. The gold standard to identify MRSA is detection of mecA by PCR, but this was not used in this study and that is a limitation. Therefore, as described above, the detection rate of MRSA in this study might be higher than actual real prevalence of MRSA. Besides, the different methods used for MRSA screening might vary across the sites. Another limitation of the study is that we observed suboptimal recording and reporting systems in some sentinel sites. The first author tried to minimize these errors by validating all the data acquired from WHONET with the registers and records of all sentinel sites. Despite this effort, we are unable to rule out data errors.

## 5. Conclusions

Myanmar has a high prevalence of MRSA infection in healthcare settings, which poses a major public health threat. Appropriate action is needed to enhance the infection control programs in healthcare settings and to focus more on the appropriate use of antibiotics. The study results offer a baseline for the future surveillance of MRSA in Myanmar and have the potential to contribute to AMR policy and stewardship. There is also a higher rate of vancomycin resistance reported in this study among *SA* isolates and this finding is alarming in the context of AMR and the accuracy of the testing needs to be validated with precise methods for confirmation.

## Figures and Tables

**Table 1:** Zone diameter in millimeter and MIC breakpoints (µg/mL) for *Staphylococcus aureus* according to CLSI guidelines.

Drug	Zone Diameter Breakpoints (mm)			MIC Breakpoints (µg/mL)		
	S	I	R	S	I	R
First-Line Drugs						
Cefoxitin	≥22	-	≤21	≤4	-	≥8
Oxacillin	-	-	-	≤2	-	≥4
Penicillin	≥29	-	≤28	≤0.12	-	≥0.25
Clindamycin	≥21	15–20	≤14	≤0.5	1–2	≥4
Erythromycin	≥23	14–22	≤13	≤0.5	1–4	≥8
Cotrimoxazole	≥16	11–15	≤10	<2/38	-	≥4/76
Nitrofurantoin	≥17	15–16	≤14	≤32	64	≥128
Second-Line Drugs						
Linezolid	≥21	-	≤20	≤4	-	≥8
Tetracycline	≥19	15–18	≤14	≤4	8	≥16
Vancomycin	-	-	-	≤2	4–8	≥8
Rifampicin	≥20	17–19	≤16	≤1	2	≥4
Gentamicin	≥15	13–14	≤12	≤4	8	≥16
Ciprofloxacin	≥21	16–20	≤15	≤1	2	≥4
Levofloxacin	≥19	16–18	≤15	≤1	2	≥4

**Table 2:** Antibiotic susceptibility pattern of *Staphylococcus aureus* isolated in seven AMR Sentinel Sites, Myanmar, 2018–2019.

Antibiotic Name (n)	Sensitive		Intermediate		Resistant	
	n	(%)	n	(%)	n	(%)
**First-line drugs**						
Cefoxitin (n = 2753)	1422	(52)	0	(0)	1331	(48)
Penicillin (n = 1302)	35	(3)	0	(0)	1267	(97)
Clindamycin (n = 2755)	1710	(62)	112	(4)	933	(34)
Erythromycin (n = 2704)	1211	(45)	206	(8)	1287	(48)
Cotrimoxazole (n = 2678)	1565	(58)	39	(1)	1074	(40)
Nitrofurantoin (n = 1332)	1287	(97)	12	(1)	33	(2)
**Second-line drugs**						
Linezolid (n = 2624)	2375	(91)	0	(0)	249	(9)
Tetracycline (n = 1426)	833	(58)	6	(0)	587	(41)
Vancomycin (n = 1249)	1124	(90)	30	(2)	95	(8)
Rifampicin (n = 1258)	1032	(82)	54	(4)	172	(14)
Gentamicin (n = 2754)	1816	(66)	79	(3)	859	(31)
Ciprofloxacin (n = 1409)	1006	(71)	46	(3)	357	(25)
Levofloxacin (n = 2765)	1812	(66)	68	(2)	885	(32)

** organism which is resistant to cefoxitin disc (30 µg) or oxacillin MIC (≥4 µg/mL) were listed as MRSA.

**Table 3:** Demographic and clinical characteristics of patients whose isolates tested positive for *Staphylococcus aureus* infection in seven AMR Sentinel Sites, Myanmar, 2018–2019.

Variable	Patients Whose Isolates Were Tested Positive with *SA* Infection (2018, n = 1324)		Patients Whose Isolates Were Tested Positive with *SA* Infection (2019, n = 1477)	
	n	(%)	N	(%)
**Age (years)**				
<15	274	(21)	284	(19)
15–44	502	(38)	545	(37)
45–64	363	(27)	448	(30)
≥65	173	(13)	186	(13)
Unknown	12	(1)	14	(1)
**Gender**				
Male	711	(54)	802	(54)
Female	609	(46)	672	(46)
Unknown	4	(0)	3	(0)
**Type of Patient**				
Inpatient	1046	(79)	1324	(90)
Outpatient	229	(17)	101	(7)
Unknown	49	(4)	52	(4)
**Type of Specimen**				
Blood	282	(21)	474	(32)
Sputum/Respiratory	149	(11)	172	(12)
Urine	107	(8)	105	(7)
Wound (pus/swab)	664	(50)	647	(44)
Body fluid	30	(2)	8	(1)
Miscellaneous	92	(7)	71	(5)
**Source of patient**				
Medical	370	(28)	472	(32)
Surgical	443	(33)	494	(33)
Paediatric	55	(4)	150	(10)
ICU	61	(5)	57	(4)
Dermatology	133	(10)	99	(7)
Emergency/OPD	94	(7)	81	(5)
Unknown	89	(7)	80	(5)
Others	79	(6)	44	(3)

**Table 4:** Prevalence of *Staphylococcus aureus* and Methicillin-resistant *Staphylococcus aureus* isolated in seven AMR sentinel sites, Myanmar, 2018–2019.

Sites	2018							2019						
	Total Isolates	Gram-Positive (n, %)		*SA* (n, %)		MRSA (n, %)		Total Isolates	Gram-Positive (n, %)		*SA* (n, %)		MRSA (n, %)	
Site A	1773	398	(22)	87	(22)	23	(26)	1388	287	(21)	56	(20)	18	(32)
Site B	5028	1570	(31)	298	(19)	114	(38)	5484	1877	(34)	348	(19)	124	(36)
Site C	1355	182	(13)	48	(26)	20	(42)	1279	228	(18)	71	(31)	17	(24)
Site D	4088	629	(15)	186	(30)	78	(42)	3378	625	(21)	187	(27)	89	(48)
Site E	2967	278	(9)	140	(50)	55	(39)	2880	391	(14)	106	(27)	23	(22)
Site F	3294	628	(19)	508	(81)	388	(76)	3371	796	(24)	607	(76)	359	(59)
Site G	480	110	(23)	57	(52)	10	(18)	1033	175	(17)	102	(58)	13	(13)
Total	18,985	3795		1324		688		18,813	4397		1477		643	

**Table 5:** Demographic and clinical factors of patients whose isolates were associated with MRSA infection in seven AMR sentinel sites, Myanmar, 2018–2019.

Variable	Patients with SA Infection (N = 2801)	Patients with MRSA Infection (N = 1331)		Prevalence Ratio (95% CI)		Adjusted PR (95% CI)		*p*-Value
	n	n	(%)					
**Year**								
2018	1324	688	(52)	Ref		Ref		
2019	1477	643	(44)	0.84	(0.78–0.91)	0.86	(0.80–0.92)	<0.001
**Age (years)**								
<15	558	256	(46)	Ref		Ref		
15–44	1047	482	(46)	0.99	(0.89–1.11)	1.15	(1.02–1.31)	0.021
45–64	811	399	(49)	1.06	(0.94–1.19)	1.19	(1.04–1.35)	<0.001
≥65	359	185	(52)	1.11	(0.97–1.27)	1.27	(1.09–1.47)	0.001
Unknown	26	9	(35)	0.73	(0.43–1.25)	0.80	(0.46–1.40)	0.451
**Gender**								
Male	1513	692	(46)	0.92	(0.85–1.00)	0.92	(0.86–0.99)	0.034
Female	1281	634	(49)	Ref				
Unknown	7	5	(71)	1.42	(0.88–2.27)	1.86	(0.91–3.80)	0.144
**Type of Patient**								
Inpatient	2370	1105	(47)	Ref		Ref		
Outpatient	330	185	(56)	1.22	(1.10–1.36)	1.07	(0.93–1.24)	0.296
Unknown	101	41	(41)	0.85	(0.67–1.08)	0.86	(0.66–1.11)	0.265
**Type of Specimen**								
Blood	756	390	(52)	Ref		Ref		
Sputum/Respiratory	321	172	(54)	1.03	(0.91–1.16)	1.00	(0.89–1.13)	0.870
Urine	212	143	(68)	1.35	(1.20–1.50)	1.21	(1.08–1.36)	0.001
Wound (pus/swab)	1311	524	(40)	0.78	(0.71–0.86)	0.98	(0.88–1.09)	0.754
Body fluid	38	17	(45)	0.86	(0.60–1.23)	0.77	(0.53–1.11)	0.164
Miscellaneous	163	85	(52)	1.02	(0.87–1.20)	1.15	(0.97–1.35)	0.088
**Source of Patient**								
Medical	842	413	(49)	Ref		Ref		
Surgical	937	353	(38)	0.77	(0.70–0.86)	0.83	(0.75–0.94)	0.003
Paediatric	205	103	(50)	1.02	(0.88–1.19)	0.87	(0.73–1.05)	0.154
ICU	118	87	(74)	1.49	(1.31–1.69)	1.16	(1.03–1.30)	0.013
Dermatology	232	153	(66)	1.34	(1.19–1.50)	2.04	(1.73–2.40)	<0.001
OPD/Emergency	175	90	(51)	1.06	(0.90–1.24)	0.91	(0.74–1.11)	0.388
Unknown	169	54	(32)	0.67	(0.53–0.84)	1.13	(0.79–1.61)	0.487
Others	123	78	(63)	1.32	(0.14–1.53)	1.17	(1.01–1.36)	0.037
**Laboratory Sites**								
Site F	1115	747	(67)	Ref		Ref		
Site A	143	41	(29)	0.44	(0.34–0.57)	0.38	(0.25–0.59)	<0.001
Site B	646	238	(37)	0.55	(0.49–0.61)	0.41	(0.36–0.47)	<0.001
Site C	119	37	(31)	0.46	(0.35–0.60)	0.47	(0.35–0.62)	<0.001
Site D	373	167	(45)	0.66	(0.58–0.74)	0.56	(0.49–0.65)	<0.001
Site E	246	78	(32)	0.47	(0.39–0.57)	0.49	(0.41–0.60)	<0.001
Site G	159	23	(15)	0.24	(0.16–0.35)	0.28	(0.19–0.41)	<0.001

## Data Availability

The data that support the findings of this study are available from the corresponding author, upon request.
